# Spectrofluorimetric Method for Determination of Citalopram in Bulk and Pharmaceutical Dosage Forms

**DOI:** 10.4103/0250-474X.45407

**Published:** 2008

**Authors:** S. G. Vasantharaju, S. Lakshmana Prabu, A. Jacob

**Affiliations:** Dept of Pharmaceutical Quality Assurance, Manipal College of Pharmaceutical Sciences, Manipal-576 104, India

**Keywords:** Citalopram, fluorimetry, excitation, emission

## Abstract

A simple accurate, sensitive reproducible spectrofluorimetric method was developed for the analysis of citalopram in pure and pharmaceutical dosage form. Citalopram showed strong native fluorescence in 0.05 M sulphuric acid having excitation at 239 nm and emission at 300 nm. All parameters like the effect of different solvents, pH, dilutions, reaction time, temperature and effect of excipients were thoroughly investigated. The calibration graph was linear in the range from 0.100 to 0.900 μg/ml. The proposed method was statistically validated and successfully applied for analysis of tablet dosage forms. The percentage recovery was found to be between 99.08% to 99.28%.

Citalopram, 1-(3-dimethylaminopropyl)-1-(4-fluorophenyl)-1,3-dihydroisobenzofuran-5- carbonitrile^1^, is one of a class of antidepressants known as selective serotonin reuptake inhibitors. It is used to treat the depression associated with mood disorders. It is also used on occasion in the treatment of body dysmorphic disorder and anxiety^2^.

A review of literature revealed that many methods such as high performance liquid chromatography and liquid chromatography^3^–^6^, for the determination of citalopram in pure, pharmaceutical preparations as well as in biological fluids have been reported. The reported methods for determination of citalopram are laborious, expensive, time consuming and require sophisticated instrumentation. The aim of the present work was to develop a new spectrofluorimetric method that is sensitive, simple, reproducible, rapid and inexpensive.

Citalopram was given as a gift sample from Torrent, Ahmedabad, India. Methanol and sulphuric acid were of AR grade, obtained from Nice Chemicals Ltd., Cochin, India. All the other reagents used were of AR grade and were procured from SD Fine Chemicals, Mumbai, India. All fluorescence measurements were done on a spectrofluorimeter RF-5301 PC (Shimadzu, Japan), with single quartz cell of 1 cm path length.

A stock solution of citalopram (500 μg/ml) was prepared. Aliquots of 500 μg/ml solution were suitably diluted with 0.05 M sulphuric acid to give the final concentration in the range of 0.100–0.900 μg/ml. The solution was scanned in the range of 200 to 500 nm against 0.05 M sulphuric acid as blank, to obtain the excitation and emission wavelength. The excitation and emission wavelength was found to be 239 nm and 300 nm, respectively.

For analysis of citalopram in tablet dosage form, two commercial brands of citalopram (20 mg strength) were procured from local pharmacy. Twenty tablets of each brand were weighed and powdered for analysis. The tablet powder equivalent to 20 mg of citalopram was weighed accurately, transferred to a clean 100 ml volumetric flask and dissolved in methanol and the final volume was made up. The solution was filtered through Whatmann filter paper No. 40. An aliquot corresponding to 0.300 μg/ml was analyzed by the method described above.

Recovery studies were done at three different levels. The pre-analyzed samples were spiked with 80%, 100% and 120% of the standard citalopram and the mixtures were reanalyzed by the proposed method. The estimation was made in triplicate. Percentage recovery was calculated from the amount of drug found in the solution.

Citalopram standard preparations were prepared in various media like buffer pH 3.0, 7.0 and 0.05 M sulphuric acid. Citalopram showed stronger native fluorescence property in 0.05 M sulphuric acid; hence it was selected as an optimum solvent for spectrofluorimetric analysis. The proposed method for determination of citalopram in tablet formulation was found to be simple, accurate, economical and rapid. Citalopram exhibited maximum excitation and emission wavelength at 239 nm and 300 nm, respectively. Linearity was shown in the concentration range of 0.100-0.900 μg/ml (y= 752.58x-13.251; correlation coefficient r^2^= 0.9989). The overlain spectra are shown in fig. 1.

**Fig. 1:**
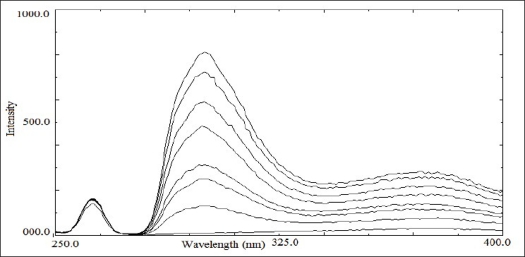
Fluorescence emission spectrum of citalopram Fluorescence emission spectrum of citalopram in 0.05 M sulphuric acid X axis depicts wavelength and Y axis depicts intensity

A relative standard deviation of 0.15% and 0.17% was observed on analysis of five replicate samples of the two brands A and B (Celica from Solus and Citara from Intas), respectively (Table 1). The percentage recovery values were between 99.08% to 99.28%. Recovery results demonstrated that the proposed method was unaffected in the presence of formulation excipients and thus highly accurate. Both inter-day as well as intra-day precision carried out, showed that the RSD is less than 1%. Results obtained confirmed ruggedness of the method. The proposed method was valid with respect to linearity, sensitivity, accuracy, reproducibility and precision. The developed method was found to be accurate, precise, reproducible and stable, which indicated that this method can be used for routine quality control of citalopram in bulk and its solid dosage form.

**TABLE 1:** ANALYSIS OF COMMERCIAL FORMULATION OF CITALOPRAM

Labeled amount (mg/tablet)	Observed amount* (mg/tablet)	% Purity
20 (Brand A)	19.94±0.03	99.70±0.15
20 (Brand A)	19.92±0.03	99.62±0.03

* Average of five readings, Values are mean±standard deviation

## References

[1] Budavari S (2007). The Merck Index.

[2] Baldessarini RJ, Beunton LL, Lazo JS, Parker KL (2006). The Pharmacological basis of therapeutics.

[3] Kristoffersen L, Bugge A, Lundanes E, Slordal L (1999). Simultaneous determination of citalopram, fluoxetine, paroxetine and their metabolites in plasma and whole blood by high-performance chromatography with ultraviolet and fluorescence detection. J Chromatogr B Biomed Sci Appl.

[4] Kosel M, Eapey M, Baumann P (1998). Analysis of the enantiomers of citalopram and its demethylated metabolites using chiral liquid chromatography. J Chromatogr B Biomed Sci Appl.

[5] Matsui E, Hoshino M, Matsui A, Okahira A (1995). Simultaneous determination of citalopram and its metabolites by high-performance liquid chromatography with column switching and fluoresence detection by direct plasma injection. J Chromatogr B Biomed Sci Appl.

[6] Oyehaug E, Ostensen ET, Salvesen B (1984). High-performance liquid chromatographic determination of citalopram and four of its metabolites in plasma and urine samples from psychiatric patients. J Chromatogr.

